# Subgroups in chronic low back pain patients – a step toward cluster-based, tailored treatment in inpatient standard care: On the need for precise targeting of treatment for chronic low back pain

**DOI:** 10.3205/000275

**Published:** 2019-09-11

**Authors:** Anna-Maria Langenmaier, Volker Eric Amelung, Matthias Karst, Christian Krauth, Franziska Püschner, Dominika Urbanski, Christine Schiessl, Reinhard Thoma, Bernhard Klasen

**Affiliations:** 1 Algesiologikum – Zentren für Schmerzmedizin, Munich, Germany; 2 Institut für Epidemiologie, Sozialmedizin und Gesundheitssystemforschung, Medizinische Hochschule Hannover, Germany; 3 Institut für Anästhesie und Intensivmedizin, Medizinische Hochschule Hannover, Germany; 4 inav – Privates Institut für angewandte Versorgungsforschung, Berlin, Germany; 5 Algesiologikum – Zentren für Schmerzmedizin, Tagesklinik für Schmerzmedizin, Munich, Germany; 6 Algesiologikum – Zentren für Schmerzmedizin, Algesiologikum MVZ Munich, Germany; 7 Algesiologikum – Zentren für Schmerzmedizin, Algesiologikum MVZ Fürth, Germany

**Keywords:** cluster analysis, chronic low back pain, multimodal treatment, inpatient routine data, distress

## Abstract

**Objective:** The purpose of this study was to find applicable clusters for the development of different treatment pathways in an inpatient multimodal pain-therapy setting based on the multifaceted nature of CLBP.

**Methods:** Based on data of questionnaires (Hospital Anxiety and Depression Scale (HADS), Marburg Questionnaire on Habitual Health Findings (MFHW), quality of life assessment using the Short-Form 12 (SF 12)), a retrospective two-step cluster analysis involving a sample of chronic low back pain (CLBP) patients (N=320) was calculated. Subsequently, the clusters were precisely described and compared on the basis of further data collected during the patients’ standard care: pain characteristics, socio-demographic data and the general state of health, psychological variables, therapy intensity, and Diagnosis Related Groups (DRG) data.

**Results:** We found a three-cluster solution: little psychological interference but marginal physical and mental quality of life (Cluster 1); poor well-being, low physical quality of life, and marginal mental quality of life (Cluster 2); and heavy mental strain and marginal physical quality of life (Cluster 3).

**Conclusions:** Similar to previous studies, our results suggest that patients suffering from CLBP differ with regard to the magnitude of mental burden and the presence of physical impairment. These differences ascertain the need for precise targeting of treatment for CLBP. Inpatient pain centers therefore should offer different multimodal therapy pathways and integrate a meaningful triage, taking into account the multifaceted nature of CLBP based on sophisticated knowledge about forms, differences, and relationships among the biopsychosocial components of CLBP.

## Zusammenfassung

**Hintergrund:** Die vorliegende Studie soll als Grundlage für die gezielte Entwicklung von Cluster-basierten Behandlungspfaden im stationären Therapiesetting mit multimodaler Schmerztherapie für Patienten mit chronischen Rückenschmerzen dienen.

**Methoden:** Basierend auf Daten aus Fragebögen (Hospital Anxiety and Depression Scale (HADS), Marburger Fragebogen zum habituellen Wohlbefinden (MFHW), Messung der Lebensqualität per Short-Form 12 (SF 12)) haben wir retrospektiv eine Two-step-Clusteranalyse unter Einbezug von N=320 Patienten mit chronischen Schmerzen berechnet. Zur anschließenden näheren Beschreibung sowie für Vergleiche zwischen den Clustern wurden Daten verwendet, die im Rahmen der stationären Regelversorgung gewonnen wurden: Schmerzcharakteristika, sozio-demografische Daten sowie Daten zum allgemeinen Gesundheitszustand, psychologische Variablen, Therapieintensität und Diagnosis Related Groups (DRG) Daten.

**Ergebnisse:** Letztendlich wurde eine Lösung mit drei Clustern gefunden: geringe psychische Beeinträchtigung, leichte Auffälligkeiten hinsichtlich körperlicher und mentaler Lebensqualität (Cluster 1); geringes allgemeines Wohlbefinden verbunden mit schlechter physischer Lebensqualität und grenzwertiger mentaler Lebensqualität (Cluster 2); und starke psychische Belastung verbunden mit grenzwertiger physischer Lebensqualität (Cluster 3).

**Schlussfolgerungen:** Ähnlich wie in früheren Studien bestätigt unsere Untersuchung, dass sich Patienten mit chronischen Rückenschmerzen hinsichtlich der psychischen Belastung und ihrer körperlichen Beeinträchtigung durchaus unterscheiden. Eben diese Unterschiede erfordern eine möglichst maßgeschneiderte Therapie für chronische Rückenschmerzen. Im Rahmen der stationären Schmerztherapie sollten daher verschiedene multimodale Behandlungspfade angeboten werden. Die Patienten könnten durch eine einfach gehaltene und gleichzeitig sinnvolle Triage, die die Vielschichtigkeit von chronischen Rückenschmerzen sowie die Unterschiede und Beziehungen der biopsychosozialen Komponenten berücksichtigt, profitieren.

## Introduction

Low back pain (LBP) is one of the leading causes of people living with disability. Thus, LBP – with its associated disability – is responsible for a significant personal burden worldwide [1]. About 70% to 80% of the Western population suffers from LBP at least once in their lifetime. A significant proportion of these patients develop chronic low back pain (CLBP) [2], which is associated with continual personal suffering and substantial economic costs [3], [4].

CLBP involves a combination of pathoanatomical, neurophysiological, physical, psychological, and social factors [5], [6], [7]. The multifaceted nature of CLBP leads to the assumption that there is no ‘general’ intervention that can target all of the underlying mechanisms and resolve its complexity [8]. It can, therefore, be assumed that it is necessary to find a classification system for CLBP which could identify the underlying mechanisms which are integrated into the biopsychosocial framework. Rusu et al. [9] defined different approaches to subgrouping CLBP patients. Responses to questionnaires are used for the empirically-derived classification of patients according to their psychosocial behavioral characteristics. Another possibility is subgrouping on the basis of the Multidimensional Pain Inventory (MPI), performed to assess physical functioning, pain severity, depressive mood, and perceived functional limitations. Further subgrouping on the basis of pain-related fear and emotional distress and focusing on the interrelationships between, for example, depressive mood and pain-related fear within individuals and across time, is possible. And finally, subgrouping on the basis of endurance responses and emotional distress with which testable hypotheses about subgroups of patients with endurance-related responses can be provided [9]. In 2010, Kent et al. proposed six phases for studies of subgroups: studies of assessment methods, hypothesis-setting studies, hypothesis-testing studies, narrow validation studies, broad validation studies, and impact analysis studies [10]. In the present study, an empirically-derived classification of patients was performed, according to their psychosocial behavioral characteristics. Furthermore, it complies with Kent et al.’s first phase as it enables relatively homogeneous subgroups to be identified while maximizing the variability between clusters [11]. With the aim of identifying patterns of patient characteristics, pain characteristics, and group-specific health care utilization [12] during the triage phase, such a classification system might enable targeted therapies and enhance the benefit to the patient significantly [13].

The idea of a classification system for LBP and CLBP is not new. Generally, both physicians and therapists agree that patients should be treated differently according to the heterogeneous conditions of LBP or CLBP [13], [14]. Many trials have been undertaken to identify subgroups, mostly with substantial differences relating to their purposes, inclusion criteria, therapeutic approaches, and methods. One purpose that plays an important role in these trials is the initial treatment of LBP and CLBP in primary care [15], [16], [17]. Other trials developed screening tools for patients’ triage, depending on the subgroups [16], [18], [19]. Another benefit of classification systems is that they might be more successful in improving treatment outcomes than global pain management strategies [20], because patients are more likely to respond to a targeted treatment method [21], [22]. Different studies have proposed various approaches to clustering patients, such as according to biological factors, mechanical loading strategies, psychological and social factors [23], [24], treatment-based factors and clinical decision-making [25], [26], [27], and factors based on pain mechanisms [28]. This list of the types of cluster analyses with their various main aims and methods is not exhaustive, but still demonstrates the diversity of approaches. The aim of the current trial is to identify homogenous subgroups of patients with CLBP within an inpatient setting. Following medical diagnosis and triage in primary care, a multimodal pain therapy was suggested for all the CLBP patients included in this study. Thus, an attempt was made to find more specific homogenous subgroups in CLBP. For this purpose, internationally validated scales were used to ensure that replicability is possible in other inpatient pain centers.

## Methods

### Study design

This trial has an empirical and exploratory character. It seeks to identify homogenous subgroups of CLBP patients in an inpatient, multimodal therapy setting; thus, it is a hypothesis-setting study [10]. The retrospective analysis of primary data [29] in the context of pain characteristics, psychological variables, socio-demographic characteristics, and treatment intensity is intended to help identify prognostic factors for a cross-sectional triage followed by graduated treatment within the inpatient, multimodal therapy setting. 

The study did not need to be referred to the ethics committee, because the data used were anonymized and the patients had already signed a consent that their data may be used for research purposes. Furthermore, it was a retrospective analysis of data which was collected during the patients’ standard care; therefore the patients did not receive placebo interventions or the like.

### Intervention

The multimodal pain-therapy setting study in which patients took part was set up as follows:

- Medical consultations (at least once per week for 30 minutes)
- Physical therapies such as physiotherapy, ergotherapy and sports for individuals and groups (for 30 minutes)
- Psychological therapy such as relaxation, and cognitive-behavioral interventions for individuals and groups (for 30 minutes)
- Pain education for groups (for 30 minutes)
- Peripheral stimulation such as TENS (as required)

### Subjects

The current dataset consists of inpatient standard care data from Algesiologikum – Centers for Pain Medicine (Munich, Germany) for patients whose initial treatment took place between 2010 and 2012 in the Algesiologikum outpatient pain center and who were subsequently targeted for a multimodal pain therapy in an Algesiologikum inpatient pain center by a specialist pain physician. The targeting resulted from medical examination, the results of the questionnaires and the doctor’s experience. The inclusion criteria were:

- Initial treatment at Algesiologikum – Centers for Pain Medicine between 2010 and 2012
- At least one back pain diagnosis (ICD-10 diagnosis: M40–M54)
- Age between 18 and 64 years, because in Germany, people of this age are usually available for the job market
- Duration of pain for at least six months
- Inpatient multimodal pain therapy for at least seven days in one of the Algesiologikum – Centers for Pain Medicine
- No missing values in the variables identified for cluster-building
- A signed declaration of consent, allowing Algesiologikum to use the data for research purposes

### Questionnaires

Before patients take part in any pain-therapy setting, they are asked to complete the German Pain Questionnaire 2007 (the current version is the German Pain Questionnaire 2015) [30], which is used in most German pain centers and includes socio-demographic information as well as internationally validated scales concerning – inter alia – biological, psychological, social, and pain mechanism-based factors. The German Pain Questionnaire 2007 comprises the following scales:

- Mainz Pain Staging System (MPSS): The assessment is based on temporal and spatial aspects, drug-taking behavior, and detailed information about the patient’s utilization of the healthcare system. The three stages of this scale represent different phases in the chronification process. The higher the stage, the more extensive are the required therapeutic interventions, and the less likely is the full recovery from chronic pain [31], [32], [33]. The MPSS’s internal consistency as well as its validity have been confirmed, for example, by Frettlöh et al. [33], Hampel et al. [34], Schmitt et al. [35], and Hüppe et al. [36].
- Chronic Pain Grade (CPG) [37], [38]: The CPG was developed to assess the severity of chronic pain problems. With seven items, it takes into consideration pain intensity (right now, worst pain in the last four weeks, pain on average in the last four weeks), the number of days in the last three months for which patients were prevented from taking part in their usual activities (work, school, housework) by pain and interference with daily activities, ability to take part in recreational, social and family activities, and ability to work (including housework) in the past three months. The four CPG values differ with respect to self-reported disability and pain intensity:
  - CPG 1: low disability and low pain intensity
  - CPG 2: low disability and high pain intensity
  - CPG 3: high disability and moderately limiting pain intensity
  - CPG 4: high disability and severely limiting pain intensity
- Klasen et al. [38] determined the reliability of CPG with an internal consistency of r=0.82 for the total scale. They verified its validity using, inter alia, MPSS and the Raspe Grading Scheme.
- Hospital Anxiety and Depression Scale (HADS): this consists of two subscales – the anxiety subscale (HADS-A) and the depression subscale (HADS-D), both of which have seven items and a range of 0–21 and a cut-off of 11. Anxiety and depression values higher than 10 must be considered to be problematic. Both subscales have been shown to be reliable and have been validated for several subgroups. The internal consistency was between 0.80 and 0.81 for both [39]. The validity of HADS has been confirmed in different studies [39], [40], [41], [42]. (In the new 2015 version of the German Pain Questionnaire, the HADS [43] has been replaced by the depression, anxiety, and stress scale (DASS); the other items used in this cluster analysis remain the same [44], [45].)
- The Marburg Questionnaire on Habitual Health Findings (MFHW): The MFHW is a seven-item scale for the assessment of the trait dimensions of well-being [46]. It has a range of 0–35 and a cut-off of 11. Well-being values lower than 11 are conspicuous. This questionnaire’s reliability is confirmed with an internal consistency between 0.87 and 0.92. Interrelations with variables indicating chronicity support the concept’s construct validity. This also applies to the stage algorithm provided by the pain clinic in Mainz, the affective dimension of the pain experience, disability, depression, and inability to work [47].
- Quality of life assessment using the Short-Form 12 (SF 12): SF 12 is the short-form of SF 36 and was developed with a total of 12 items to reproduce the Physical Component Summary (PCS; internal consistency between 0.77 and 0.93) and the Mental Component Summary (MCS; internal consistency between 0.78 and 0.88). Both the PCS and MCS range from 0 (lowest level of health measured by the scales) to 100 (highest level of health). The cut-off for the PCS is 29, while that of the MCS is 44. PCS values lower than 29 points and MCS values lower than 44 points must be considered to be problematic. In 14 validity tests involving physical criteria, relative validity estimates for the 12-item PCS ranged from 0.43 to 0.93 (median=0.67), compared to the best 36-item short-form scale. Relative validity estimates for the 12-item, MCS in 6 tests involving mental criteria ranged from 0.60 to 107 (median=0.97) in relation to the best 36-item short-form scale [48], [49].
- The List of Pain Description (Schmerzbeschreibungsliste, SBL) was developed to measure the sensory and affective aspects of pain in a differentiated manner in order to gain indicators of psychological burden. The score has a range of 0–12 and a cut-off of 9. An SBL value of 8 stands for an increased pain experience. Higher values are conspicuous. The internal consistency was confirmed with Cronbach’s alpha between 0.79 and 0.83 [50].

### Statistical analyses

The items in the full item pool (see measurements) which were most relevant for the variance explanation were identified with the k-means procedure (squared Euclidean distances) using Almo 15 [51]. By maximizing the variability between clusters, the k-means procedure also identifies relatively homogenous groups. Almo 15 provides statistical measurements for evaluating the appropriateness of a cluster solution (F-value and eta^2^) [52]. Several analyses were run with different combinations of all the included variables to identify those with the highest eta^2^ as cluster-building variables. A two-step cluster analysis with SPSS 22 was carried out using the findings of the k-means procedure. Group differences were calculated, not only for cluster-building variables, but also for other surveyed variables, in order to give an extensive impression of the patients in the clusters. Group differences for nominal- and ordinal-scaled items were identified using the chi-square test and mean differences, while post-hoc tests (Scheffé) were carried out for interval and ratio-scaled items. The chi-square test is a one-sample test which computes a chi-square value based on the differences between the observed and expected frequencies in each category. The post-hoc Scheffé test performs simultaneous joint pair-wise comparisons for every possible pair-wise combination of means, using the F sampling distribution [53]. The level of significance was set at 0.05.

The calculation of a two-step clustering algorithm does not allow any missing values. Thus, all cases with any missing values in the cluster-building variables were excluded.

The daily routine at a pain center providing basic medical care does not allow the staff to monitor patients while they are filling in questionnaires. Furthermore, some questions, such as ‘job situation’, are optional. Cases with missing values in non-cluster-building variables were included because they still have informational content. Only valid cases were used for each variable. For this reason, some of the total values may differ from the sum of the values for all included patients. Moreover, valid percentages were used in the analyses.

### Cluster-building variables

- HADS-A
- HADS-D
- MFHW
- PCS
- MCS

### Further variables

**Socio-demographic data and general state of health**

- Sex
- Age
- Working status
- Duration of CLBP
- Number of comorbidities
- Number of doctor visits before beginning treatment in the Algesiologikum

**Pain characteristics**

- MPSS
- CPG with pain intensity and interferences

**Psychological variables**

- SBL

Data regarding therapy intensity was captured with QUAST – a computer-assisted tool for documentation and quality assurance in pain treatment [54].

### Diagnosis Related Groups (DRG) data

Furthermore, the DRG data from the inpatient pain centers were contained in the §21 datasets which contain hospital-specific structural data, and case-related performance data, such as diagnoses, DRGs, admission reasons and dates, discharge reasons and dates, treatment processes, birthdates, and duration of stay. By law, each hospital has to submit its previous year’s §21 dataset to the national DRG data department by March 31 [55]. The variables taken from this source were:

- Duration of stay in hospital

### Diagnoses are grouped following the German ICD-10 codes

- F30–F39: Affective disorders
- F40–F48: Neurotic stress and somatoform disorders
- G44–G47: Episodic and paroxysmal diseases of the nervous system
- G50–G59: Diseases of nerves, nerve roots, and nerve plexus
- M40–M54: Diseases of the spinal cord and spine
- M60–M79: Diseases of soft tissues
- R50–R69: General symptoms

## Results

For the reporting period, 320 patients were found who fulfilled the inclusion criteria mentioned previously. In total, 280 of the 600 patients treated during this period were excluded prior to the two-step cluster analysis because of the following factors:

- Age older than 64 (N=146; 24.3%)
- Pain diagnosis other than back pain (N=98; 16.3%)
- Duration of pain for less than six months (N=11; 2.0%)
- Missing values in cluster-building variables (N=66; 11%)

Some of the excluded patients met several exclusion criteria.

### Participants

As indicated in Table 1 (Tab. 1), approximately 65% of the sample patients were female. The CLBP patients were on average 49 years old and had been suffering from severe CLBP (MPSS=3; CPG=4) for at least five years. About half of the patients were employed, approximately a quarter were unemployed, and a further 17% were retired.

Patients stayed in hospital for 15 days on average and participated in an average of 23 psychological and 44 physical therapy sessions. All the patients were encouraged to take responsibility for their pain management. To ensure that all biomedical and psychological aspects of pain problems are managed optimally, treatment is to be multidisciplinary and involve specialists when needed, in order to achieve the goal of reducing pain and/or improving pain management, as well as improving the patients’ physical, psychological, work and social role functioning [56].

### Description of cluster modeling

The following variables were identified as cluster-building variables (Table 2 (Tab. 2)): HADS-A, HADS-D, MFHW, PCS, and MCS.

A three-cluster solution demonstrating reasonable quality criteria was determined by using these five variables (F-value=125,070; eta^2^=0.404). These three clusters explained 40.4% of the variance.

The results of the k-means procedure excluded other variables, such as the number of interventions, SBL, age, etc., which made no meaningful contribution. In the second step, the same dataset and cluster-building variables were used for a two-step cluster analysis using SPSS 22. (Note that the cluster-feature tree and the final solution may depend on the order of cases [53]). Following the three-cluster solution which had been determined using Almo 15, a three-cluster solution was also identified for the two-step cluster analysis with SPSS 22. The quality of this solution was fair and the data also reflected weak evidence of cluster structure [57].

### Description of clusters

While only significant differences are described in the written description of the clusters below, the following tables provide a detailed characterization of the three clusters in terms of number, proportion, mean, standard deviation (SD) and range, as well as error probabilities and group comparisons: Table 3 (Tab. 3), Table 4 (Tab. 4), Table 5 (Tab. 5), Table 6 (Tab. 6), Table 7 (Tab. 7), Table 8 (Tab. 8), Table 9 (Tab. 9).

### Cluster 1 – little psychological interference but marginal physical and mental quality of life

Cluster 1 (N=72, 22.5% of all the included patients) consists of patients with moderate mental strain, who showed no conspicuous features associated with anxiety, depression, and poor psychological well-being. The PCS and MCS values were reduced but still acceptable.

Patients in Cluster 1 were significantly less depressive and had a better well-being and a better physical status than those in Cluster 2. Furthermore, they obtained better values for all cluster-building scores than those patients in Cluster 3 (Table 3 (Tab. 3)).

Cluster 1 has more employed patients (63.9%) than Cluster 2 and Cluster 3, as well as fewer unemployed patients than Cluster 3 (Table 4 (Tab. 4)).

The average and worse pain intensities were lower in Cluster 1 than in Clusters 2 and 3. Likewise, interference with daily, recreational, social, and family activities, the ability to work, and the number of days for which patients were kept from their usual activities (work, school, or housework) during the last three months were lower than in Clusters 2 and 3 (Table 6 (Tab. 6)).

In comparison to Cluster 3, patients were found in Cluster 1 with an MPSS score of I, and fewer patients with an MPSS score of III. Patients in Cluster 1 had a CPG score of 3 more often than those in Clusters 2 and 3. At the same time, the number of patients with a CPG score of 4 was lower in Cluster 1 than in Clusters 2 and 3. Concerning affective pain experience, as measured with SBL, it was found that the patients in Cluster 1 were less affected than those in Cluster 3, but none of the mean scores indicated a clinically problematic state (Table 7 (Tab. 7)).

With respect to the ICD-10-diagnosis groups “Neurotic stress and somatoform disorders” (F40–F48: 45.8%) and “Diseases of nerves, nerve roots, and nerve plexus” (G50–G59: 2.8%), the patients in Cluster 1 were less frequently affected than the patients in Cluster 3 (Table 8 (Tab. 8)). The diagnoses “Depressive episode” (F32: 11.1%), “Recurrent depressive episodes” (F33: 9.7%), and “Somatoform disorders” (F45: 37.5%), were less prevalent in Cluster 1 than in Cluster 3 (Table 9 (Tab. 9)).

### Cluster 2 – poor well-being, low physical quality of life, and marginal mental quality of life

Cluster 2 (N=80, 25.0% of all included patients) was mostly comprised of physically affected patients (PCS<29) with a considerably lowered value for well-being (MFHW<11). Anxiety, depression and MCS showed regular values.

In comparison to the patients in Cluster 1, those in Cluster 2 were more affected by depression, poor well-being, and PCS. In addition, the patients in Cluster 2 showed worse values for PCS than those in Cluster 3. There were no differences in respect of well-being between Cluster 2 and 3. The other cluster-building variables (HADS-A, HADS-D, and MCS) were lower in Cluster 3 than in Cluster 2.

The patients in Cluster 2 were less likely to be employed (47.5%) than those in Cluster 1. In terms of working status, there were no differences between Clusters 2 and 3 (Table 4 (Tab. 4)).

The patients in Cluster 2 had higher average and worse pain intensities than the patients in Cluster 1. Cluster 2 patients also showed more interference from pain in their daily activities, recreational, social, and family activities, and ability to work than those in Cluster 1. Patients in Cluster 2 reported experiencing more days when they were kept from their usual activities (work, school, or housework) in the last three months than those in Cluster 1. No differences were found with regard to pain intensity, interference, and days kept from usual activities between Clusters 2 and 3 (Table 6 (Tab. 6)).

There were no group differences between Cluster 2 and the other clusters relating to MPSS. More patients in Cluster 2 had a CPG score of 4 and fewer had a CPG score of 3 – in contrast to those in Cluster 1. In respect of CPG, no differences between Clusters 2 and 3 were found. The values for affective pain experience (SBL) were similar between Clusters 2 and 1, as well as between Clusters 2 and 3 (Table 7 (Tab. 7)).

Cluster 2 had fewer patients with diagnoses from the diagnosis group “Episodic and paroxysmal diseases of nervous system” (G44–G47 16.3%) than Cluster 3, as shown in Table 8 (Tab. 8). Regarding “Recurrent depressive episodes” (F32 7.5%), the patients in Cluster 2 were less affected than those in Cluster 3 (Table 9 (Tab. 9)).

### Cluster 3 – heavy mental strain and marginal physical quality of life

The patients in Cluster 3 (N=168, 52.5% of all included patients) showed clinically significant values for all the cluster-building variables.

In comparison to Cluster 1, differences were found in each cluster-building variable. Overall, while the patients in Cluster 3 showed lower values for psychological mood variables, the patients in Cluster 2 suffered more in terms of physical components.

The patients in Cluster 3 were less likely to be employed (45.1%) than those in Cluster 1. Cluster 3 had more unemployed patients (29.9%) than Cluster 1. In respect of working status, no differences were found between Clusters 3 and 2 (Table 4 (Tab. 4)).

The actual, average, and worst pain intensities were higher when compared to Cluster 1. Likewise, interference due to pain in daily, recreational, social, and family activities, the ability to work, and number of days on which patients were kept from their usual activities (work, school, or housework) during the last three months were higher than in Cluster 1. In regard to pain intensity, interference, and days kept from usual work, no differences were found between Clusters 2 and 3 (Table 6 (Tab. 6)).

In contrast to Cluster 1, no patients were found with an MPSS value of I in Cluster 3, but there were significantly more patients with an MPSS value of III. Fewer patients in Cluster 3 had CPG scores of 2 and 3 compared to those in Cluster 1. However, there were more patients with a CPG score of 4 in Cluster 3 than in Cluster 1. Patients in Cluster 3 had a lower SBL value than those in Cluster 1. There was no difference in terms of MPSS, CPG, or SBL between Clusters 2 and 3 (Table 7 (Tab. 7)).

With regard to the diagnosis groups “Neurotic stress and somatoform disorders” (F40–F48: 63.1%) as well as “Diseases of nerves, nerve roots, and nerve plexus” (G50–G59: 10.7%), the patients in Cluster 3 were more affected than those in Cluster 1. The patients in Cluster 3 suffered from “Episodic and paroxysmal diseases of nervous system” (G44–G59: 29.2%) more often than those in Cluster 2 (Table 8 (Tab. 8)). The diagnoses “Depressive episode” (F32: 30.4%) and “Somatoform disorders” (F45: 57.1%) were more prevalent in Cluster 3 than in Cluster 1. The incidence of “Recurrent depressive episodes” (F33: 22.0%) was higher in Cluster 3 than in Clusters 1 and 2 (Table 9 (Tab. 9)).

## Discussion

In this study, the aim was to draw attention to the need for inpatient multimodal treatment for patients suffering from severe CLBP and who had already been under treatment for a long time, to be precisely targeted. The aim was to find homogenous subgroups among those CLBP patients for whom an inpatient multimodal pain therapy was recommended following medical diagnosis and triage in primary care. The fact that no differences were observed for other variables such as “duration of CLBP” (Table 8 (Tab. 8)), “number of doctor visits” (Table 5 (Tab. 5)) before starting the multimodal treatment, and “number of co-morbidities” (Table 5 (Tab. 5)) could be an evidence for an at least similar form of targeting in primary care.

It was hypothesized that because CLBP is so multifaceted, there was a need to find more sophisticated subgroups to be targeted, more individualized treatment, and a better quality of care, especially in the inpatient treatment sector. Further targeting might improve inpatient multimodal pain therapy by making it more individualized and effective for different groups. For this purpose, international validated scales were used so that replicability might be possible in other inpatient pain centers and a two-step cluster analysis of those CLBP patients who were targeted to multimodal pain therapy was calculated.

As the factors “anxiety”, “depression”, “well-being”, “physical component summary”, and “mental component summary” explained 40.4% of the variance, they were identified as cluster-building variables, and a meaningful three-cluster differentiation was found. The groups identified could be characterized as “little psychological interference but marginal physical and mental quality of life”, “poor well-being, low physical quality of life, and marginal mental quality of life”, and “heavy mental strain and marginal physical quality of life.”

The differences concerning non-cluster-building variables could help the therapists obtain a global idea of the patients in the different clusters: Especially their mental and physical status, but also their information regarding pain intensity, days kept from usual activities, interference with daily activities, ability to take part in recreational, social, and family activities, ability to work (including housework), CPG, MPSS, SBL, therapy intensity, diagnoses groups, diagnoses, and working status could help achieve an individual focus for group-related, inpatient therapy pathways.

The Cluster 1 patients seemed to be reasonably healthy in contrast to the patients in Clusters 2 and 3. They had chronic pain but they were not very affected in terms of its physical or psychological aspects. The therapeutic focus for this group should be on pain relief or coping strategies, in order to avoid further chronification.

Compared to the patients in Cluster 3, those in Cluster 2 were healthier in terms of every cluster-building variable with the exception of MFHW, but when compared to those in Cluster 1, they seemed to be more affected. They had primarily physical symptoms which had a bad influence on their general well-being. In order to ease these symptoms and to improve patient’ physical strength and general well-being, a therapy focus on exercise would be necessary for this group.

The patients in Cluster 3 seemed to suffer more from their CLBP than those in Clusters 1 and 2, especially in terms of mental strain. The main focus of therapy for these patients should be a psychological approach, because they seemed to be highly distressed.

Homogenous subgroups among those patients suffering from CLBP, based on the patients’ clinical presentations, might improve treatment outcomes [20], [58]. The clusters were formed based on internationally-validated scales to reduce subjectivity and make this study transferable to other pain centers [10]. Several studies using different approaches to search for subgroups among CLBP patients were found. Solovieva et al. [59], Tegeder et al. [60], Karpinnen et al. [61], Costigan et al. [62] for example, found subgroups based on genetic factors, while Turk et al. [63], Turk [64], Dahlstrom et al. [65], Turk et al. [66], Johansson et al. [67], Turk et al. [68], Soderlund et al. [69], Thieme et al. [70], Widerström-Noga et al. [71], Heidari et al. [72] found subgroups based on psychosocial factors, and Huijen et al. [73], McCracken et al. [74], Hasenbring et al. [75] based subgroups on activity-related behavior. As all CLBP patients had a back pain diagnosis in common, it seemed logical to cluster these patients based on their pain characteristics as well as psychological and social factors, as was done by Waddell [7], Hasenbring et al. [75], Foster [76], Kamper [77], and Wettstein et al. [78] in various previous cluster analyses.

The aim was to demonstrate the need for the precise targeting of primary care treatment of CLBP patients, as was done, for example, by Viniol et al. [79] who identified three clusters consisting of “pensioners with age-associated pain caused by degenerative diseases”, “middle-aged patients with high mental distress and poor coping resources”, and “middle-aged patients who are less affected by pain and better positioned with regard to mental health”. Boersma et al. [80] found four similar group profiles: “fear avoidant”, “distressed fear avoidant”, “low risk”, and “low-risk depressed mood”. Hirsch et al. [12] also identified four clusters in primary care: “elderly patients adapted to pain”, “patients with chronic severe pain with comorbid depression”, “younger patients with subacute pain and emotional distress”, and “younger patients with acute pain”. Wettstein et al. [78] identified three distinct well-being profiles, characterized by “generally high well-being”, “moderate well-being”, and “consistently low well-being”, respectively. The results of the current study reaffirm those of the previous cluster analyses, in particular in respect of the individual, multifaceted nature of CLBP.

The profiles taken into consideration range from very distressed patient groups who were hardly able to take part in daily social activities, over groups consisting of adaptive patients who could cope and hence experienced less pain, were less distressed, and were able to take part in daily social activities, through to groups of patients who were experiencing severe pain and high levels of distress, but still taking part in daily social activities.

While the focus of this study was on improving the triage of CLBP patients from different groups, Hill et al. [81], for example, tested the outcome of differentiated treatment. They compared the clinical effectiveness and cost-effectiveness of stratified primary care (intervention) with the non-stratified, current best practice (control). At twelve months, the stratified care was associated with a mean increase in generic health benefits and cost savings compared to the control group [81].

Fehrmann et al. [82] took a different approach, inspired by Hasenbring et al.’s avoidance-endurance model [83], which recommends subgroup-tailored interventions – confronting fear-avoidant patients with their fear of movement and encouraging them to stay active, whereas endurers should be encouraged to avoid overexerting themselves and to take breaks in time. Fehrmann et al. [82] sought to examine whether or not the avoidance-endurance model subgroups, fear avoiders (FAR), distress endurers (DER), eustress endurers (EER), and adaptive responders (AR), showed differences in physical measures and outcomes after training therapy and found that the DER and the FAR groups were more impaired before and after the intervention, compared with EER and AR, as indicated by a higher pain intensity, higher disability levels, lower quality of life, and inferior working capacity.

These findings encourage the authors to also verify the benefits of stratified care for the “types” of patients with CLBP identified in this study, in further investigation.

It was hypothesized that CLBP patients would differ in terms of therapy intensity, due to them probably having different needs, but there were no differences in therapy intensity. A reason for this might be that patients with a higher level of fitness or rather less mental strain participated more frequently. Another reason might be that, in the case of multimodal pain therapy, the DRG system does not provide incentives to provide high-intensity therapy because the payments do not increase for higher performance in terms of therapy intensity. On the contrary, the higher the therapy intensity offered, the lower the pain center’s profit. The integration of more clinical considerations, such as adjusting therapy intensity to individual needs, can be more economical while simultaneously improving the quality of a therapy. Inpatient pain centers should offer different multimodal therapy pathways for effective treatment. Therefore, the development of a suitable algorithm which targets patients early so that they receive optimal care as soon as possible is essential. At least two of the three clusters in this study need highly intensive and expensive pain therapy. The difficulty does not lie in developing and offering such programs, but in convincing health insurance providers that certain patients need such high-intensity pain therapy. This trial should also be a basis for the corresponding arguments about which patients fit which treatment pathway, including the required therapy intensity.

This study has shown that it is next to impossible to carry out a meaningful triage of patients with severe CLBP without sophisticated knowledge about its forms as well as differences and relationships between the biopsychosocial components. The benefits of stratified care for patients are tailored treatment, individual therapeutic goals, and a higher quality of life. Care providers might profit from it because they would be able to specialize their infrastructure, become more (cost-)effective, and achieve better therapy outcomes while simultaneously increasing revenues. Employers and colleagues could also benefit from stratified care, because employees suffering from CLBP might need fewer days off from work after the specialized pain therapy. Although highly intensive pain therapy is associated with higher costs, the costs related to these patients might come down in the long term, because a better therapy outcome could lead to less medication being required, fewer doctor’s visits, fewer stays in hospital, fewer days off work, and perhaps even the ability to work again. Moreover, it is assumed that stratified care creates the space needed to develop and conclude individual, special treatment pathways, contracts between health insurance providers, non-state actors in the healthcare system, and care providers. Furthermore, an assumption for the future is that stratified care will lead to fewer days off work which enhances economic efficiency and productivity, as well as possibly avoiding costs relating to early retirements, which would have positive implications for the economy [77]. Therefore, stratified care should be a common aim for all therapists as well as for all actors in health insurance, the healthcare system, and for other providers involved in CLBP treatment.

The differences found in the current study not only caused CLBP patients to be targeted into special kinds of pain treatment or care, such as inpatient or outpatient. They caused an individual kind of multimodal pain therapy to be developed for each separate CLBP group by taking into account the heterogeneous and multifactorial nature of CLBP. It is hoped that this study will be understood as a form of support, or rather an incentive, for other providers of multimodal pain therapy, in order to develop individual treatment pathways for their CLBP patients’ different needs.

## Limitations

The present study has some limitations that need to be considered. The first point that should be mentioned is that cluster analysis is a useful technique for revealing an inherent structure in a dataset, but it must be recognized that it will impose a structure on any dataset, regardless of whether or not one exists. For this reason, no one cluster solution found is likely to be entirely valid for a given pain population and should always be cross-validated [74]. The absence of cross-validation with another pain center or broader range of patient types is another limitation. Therefore, it would be advisable to examine this study in other circumstances, especially relating to the targeted treatment pathways which are currently being tested. In addition, the results of a cluster analysis depend on the input variables [84]. The strength of this evaluation is that internationally-validated scales were used. However, if even one questionnaire were to be unavailable, this model would no longer be transferable.

Furthermore, it is a retrospective trial using routine basic medical care data because the study design was not specified before the evaluation and the day-to-day business kept running as usual. In addition, the pain center staff was not trained with regard to monitoring questionnaires. Especially in the case of some non-cluster-building variables, there were a lot of missing values and therefore also exclusions. In spite of missing values in some variables, there was still informative content that could provide a better idea of the patient’s needs.

With regard to therapy intensity, a randomized controlled trial would be necessary to detect “dose-response relationships”: Unfortunately, such randomized controlled trials are hardly possible in a standard care setting.

## Notes

### Competing interests

The authors declare that they have no competing interests. Conflicts might arise if the results of this study lead to a change in the revenue situation in any particular direction.

## Figures and Tables

**Table 1:**
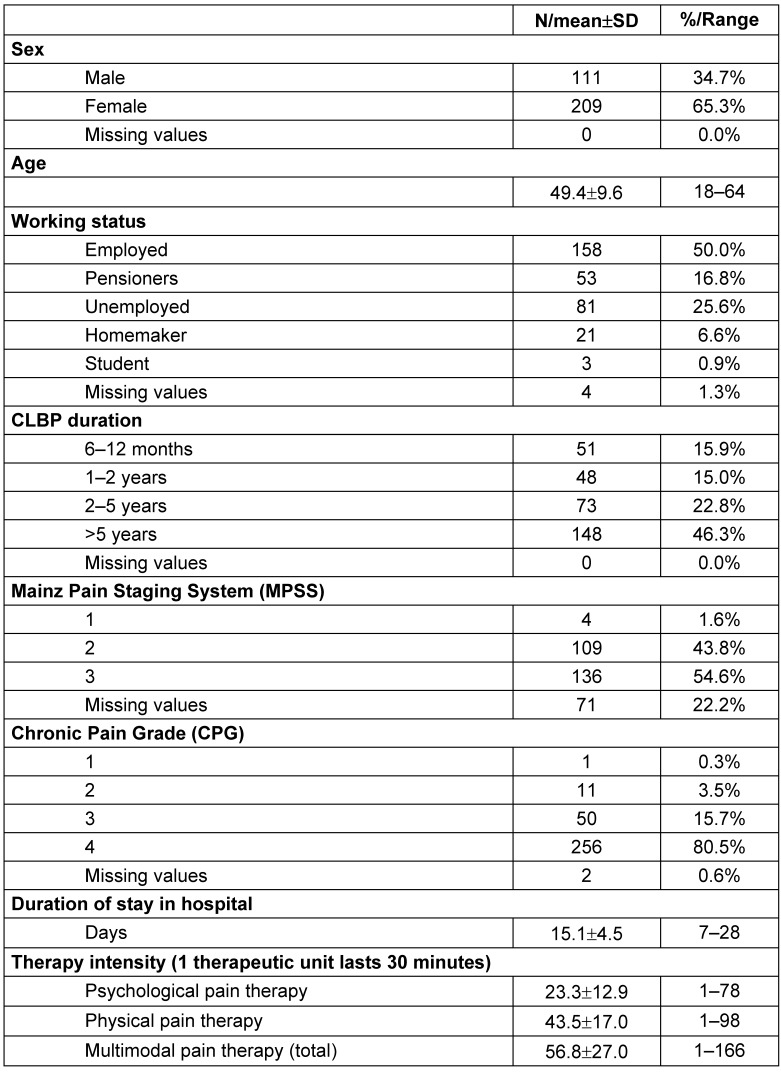
Socio-demographic data and pain impairment of the analyzed patients (N=320) (N: Number; SD: standard deviation)

**Table 2:**
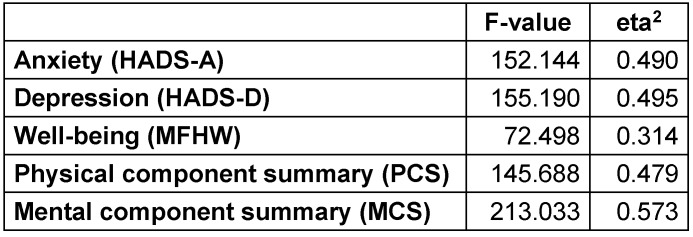
Cluster-building variables’ contribution to the separation of clusters in Almo 15 (N=320)

**Table 3:**
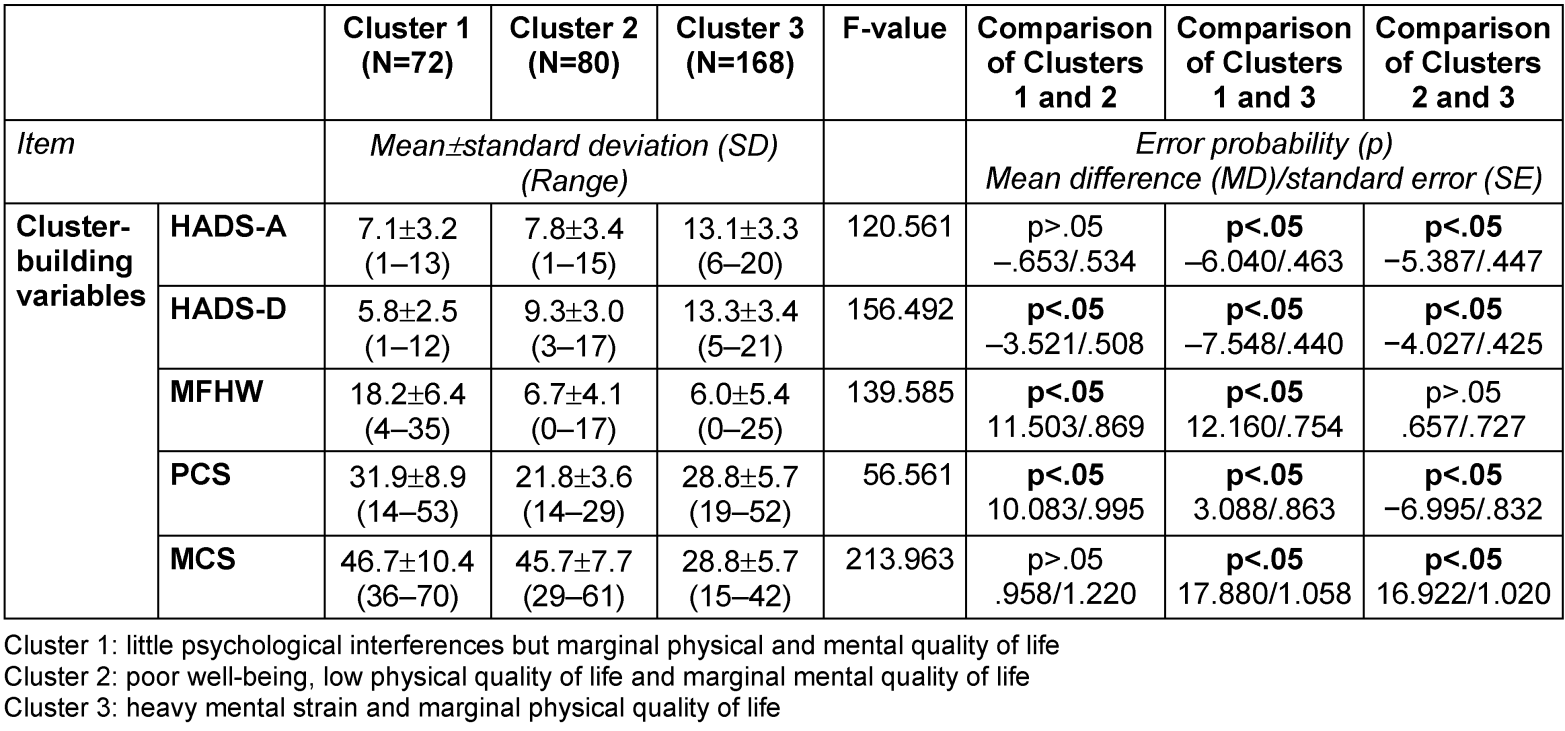
Characterization of the three clusters calculated with SPSS 21 (N=320): cluster-building variables

**Table 4:**
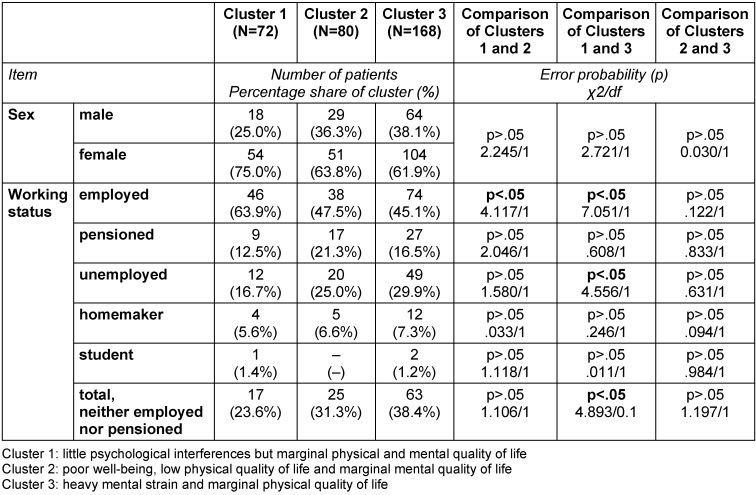
Characterization of the three clusters calculated with SPSS 21 (N=320): socio-demographic data

**Table 5:**
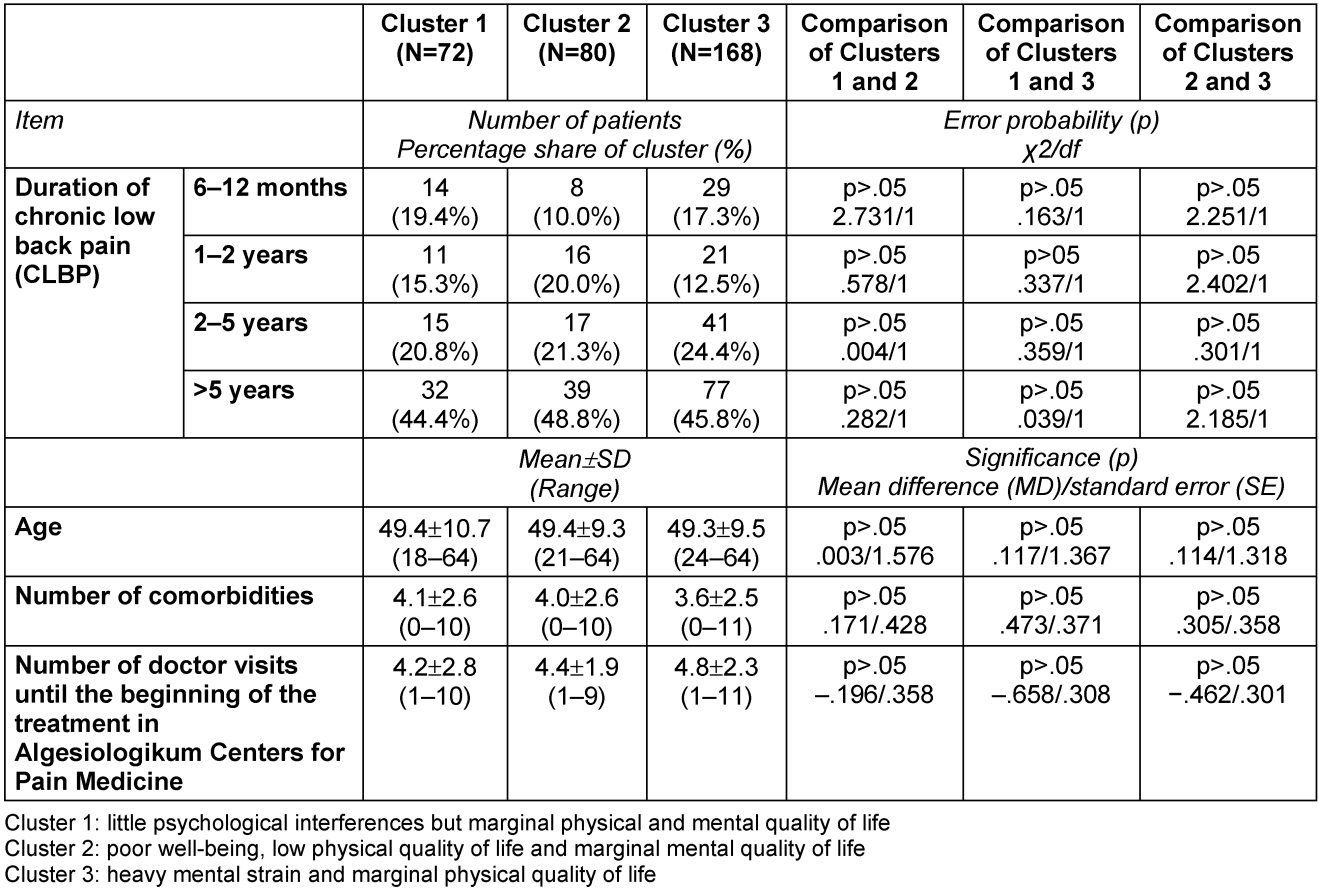
Characterization of the three clusters calculated with SPSS 21 (N=320): general state of health and age

**Table 6:**
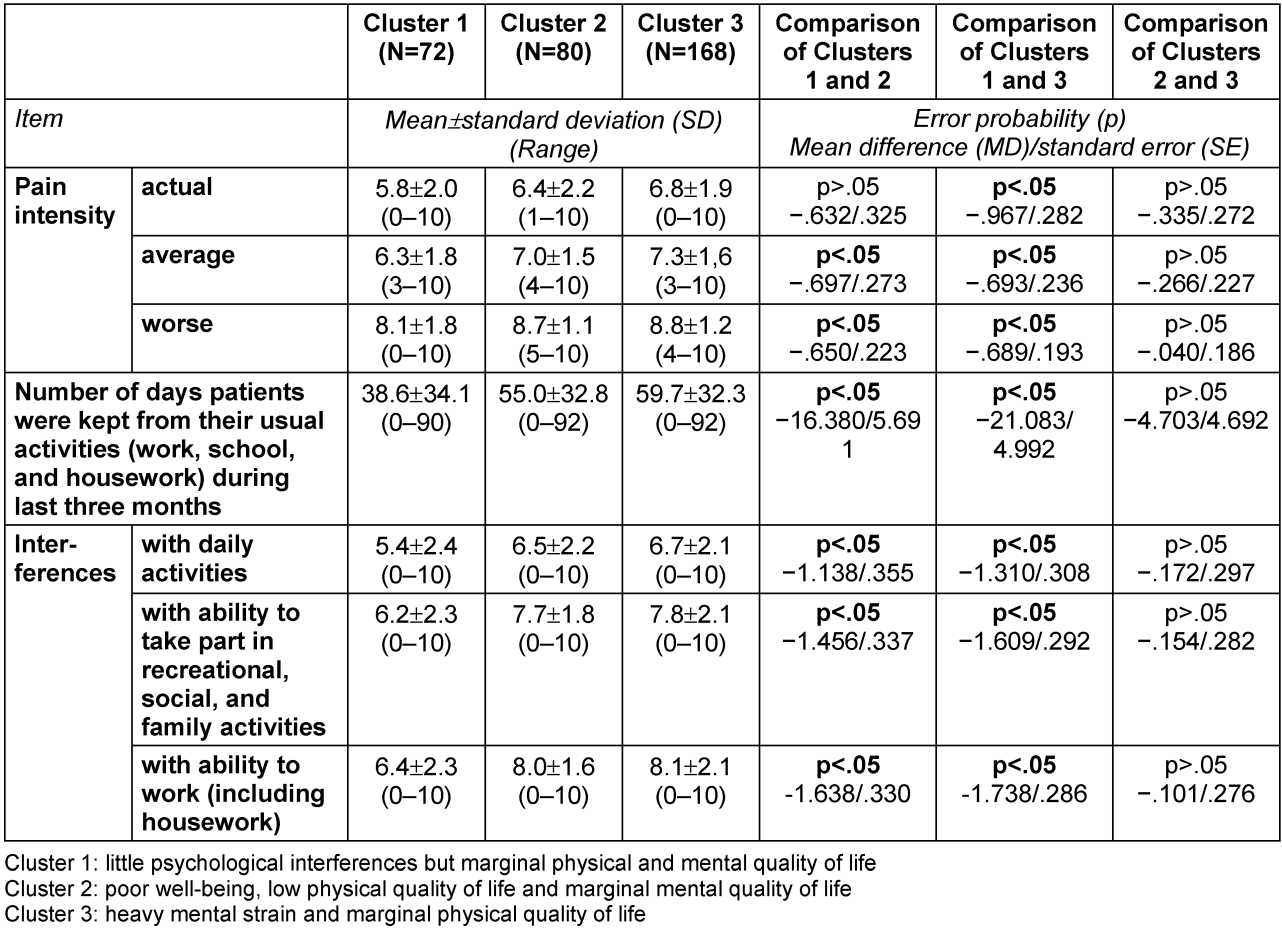
Characterization of the three clusters calculated with SPSS 21 (N=320): pain characteristics I

**Table 7:**
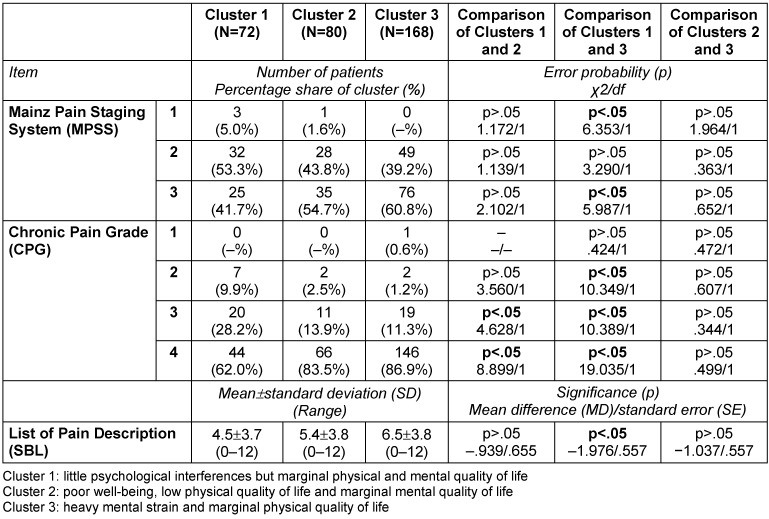
Characterization of the three clusters calculated with SPSS 21 (N=320): pain characteristics II and qualitative pain characterization (SBL)

**Table 8:**
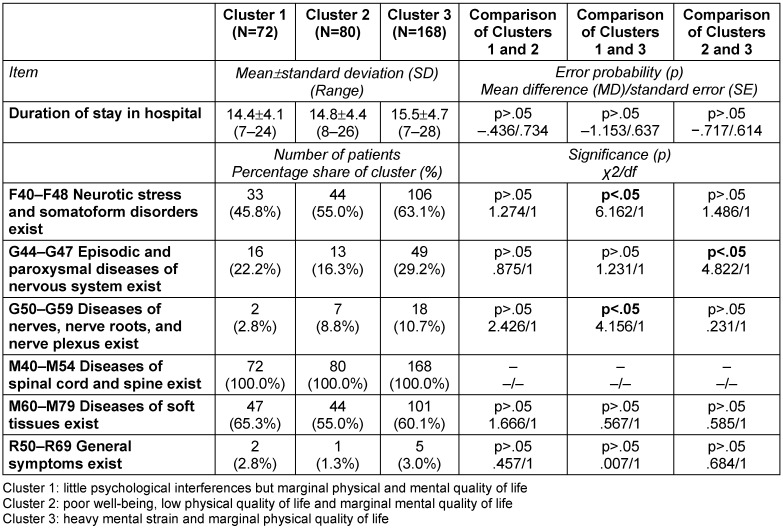
Characterization of the three clusters calculated with SPSS 21 (N=320): duration of stay in hospital and diagnosis groups

**Table 9:**
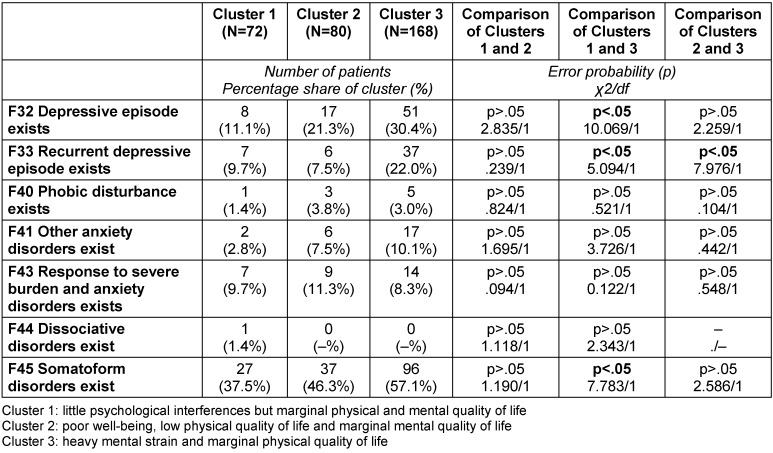
Characterization of the three clusters calculated with SPSS 21 (N=320): psychical diagnosis

## References

[1] Vos T, Flaxman AD, Naghavi M, Lozano R, Michaud C, Ezzati M (2012). Years lived with disability (YLDs) for 1160 sequelae of 289 diseases and injuries 1990–2010: a systematic analysis for the Global Burden of Disease Study 2010. Lancet.

[2] Henschke N, Maher CG, Refshauge KM, Herbert RD, Cumming RG, Bleasel J, York J, Das A, McAuley JH (2008). Prognosis in patients with recent onset low back pain in Australian primary care: inception cohort study. BMJ.

[3] Lambeek LC, van Tulder MW, Swinkels IC, Koppes LL, Anema JR, van Mechelen W (2011). The trend in total cost of back pain in The Netherlands in the period 2002 to 2007. Spine (Phila Pa 1976).

[4] Maetzel A, Li L (2002). The economic burden of low back pain: a review of studies published between 1996 and 2001. Best Pract Res Clin Rheumatol.

[5] McCarthy CJ, Arnall FA, Strimpakos N, Freemont A, Oldham JA (2004). The Biopsychosocial Classification of Non-Specific Low Back Pain: A Systematic Review. Phys Ther Rev.

[6] Borkan J, Van Tulder M, Reis S, Schoene ML, Croft P, Hermoni D (2002). Advances in the field of low back pain in primary care: a report from the fourth international forum. Spine (Phila Pa 1976).

[7] Waddell G (2004). The Back Pain Revolution.

[8] Fersum KV, Dankaerts W, O’Sullivan PB, Maes J, Skouen JS, Bjordal JM, Kvåle A (2010). Integration of subclassification strategies in randomised controlled clinical trials evaluating manual therapy treatment and exercise therapy for non-specific chronic low back pain: a systematic review. Br J Sports Med.

[9] Rusu AC, Boersma K, Turk DC, Hasenbring MI, Rusu AC, Turk DC (2012). Reviewing the concept of subgroups in subacute and chronic pain and the potential of customizing treatments. From acute to chronic back pain: Risk factors, mechanisms, and clinical implications.

[10] Kent P, Keating JL, Leboeuf-Yde C (2010). Research methods for subgrouping low back pain. BMC Med Res Methodol.

[11] Bacher J, Pöge A, Wenzig K (2010). Clusteranalyse: Anwendungsorientierte Einführung in Klassifikationsverfahren.

[12] Hirsch O, Strauch K, Held H, Redaelli M, Chenot JF, Leonhardt C, Keller S, Baum E, Pfingsten M, Hildebrandt J, Basler HD, Kochen MM, Donner-Banzhoff N, Becker A (2014). Low back pain patient subgroups in primary care: pain characteristics, psychosocial determinants, and health care utilization. Clin J Pain.

[13] O’Sullivan P (2005). Diagnosis and classification of chronic low back pain disorders: maladaptive movement and motor control impairments as underlying mechanism. Man Ther.

[14] Kent P, Keating J (2004). Do primary-care clinicians think that nonspecific low back pain is one condition? Spine (Phila Pa 1976).

[15] Foster NE, Hill JC, Hay EM (2011). Subgrouping patients with low back pain in primary care: are we getting any better at it?. Man Ther.

[16] Hill JC, Dunn KM, Lewis M, Mullis R, Main CJ, Foster NE, Hay EM (2008). A primary care back pain screening tool: identifying patient subgroups for initial treatment. Arthritis Rheum.

[17] Lacey RJ, Strauss VY, Rathod T, Belcher J, Croft PR, Natvig B, Wilkie R, McBeth J (2015). Clustering of pain and its associations with health in people aged 50 years and older: cross-sectional results from the North Staffordshire Osteoarthritis Project. BMJ Open.

[18] Murphy SE, Blake C, Power CK, Fullen BM (2016). Comparison of a Stratified Group Intervention (STarT Back) With Usual Group Care in Patients With Low Back Pain: A Nonrandomized Controlled Trial. Spine (Phila Pa 1976).

[19] Verra ML, Angst F, Staal JB, Brioschi R, Lehmann S, Aeschlimann A, de Bie RA (2012). Reliability of the Multidimensional Pain Inventory and stability of the MPI classification system in chronic back pain. BMC Musculoskelet Disord.

[20] Billis E, McCarthy CJ, Gliatis J, Gittins M, Papandreou M, Oldham JA (2012). Inter-tester reliability of discriminatory examination items for sub-classifying non-specific low back pain. J Rehabil Med.

[21] Fritz JM, George S (2000). The use of a classification approach to identify subgroups of patients with acute low back pain. Interrater reliability and short-term treatment outcomes. Spine (Phila Pa 1976).

[22] Hicks GE, Fritz JM, Delitto A, McGill SM (2005). Preliminary development of a clinical prediction rule for determining which patients with low back pain will respond to a stabilization exercise program. Arch Phys Med Rehabil.

[23] Hancock MJ, Maher CG, Laslett M, Hay E, Koes B (2011). Discussion paper: what happened to the ‘bio’ in the bio-psycho-social model of low back pain?. Eur Spine J.

[24] Pincus T, Burton AK, Vogel S, Field AP (2002). A systematic review of psychological factors as predictors of chronicity/disability in prospective cohorts of low back pain. Spine (Phila Pa 1976).

[25] Hebert JJ, Koppenhaver SL, Walker BF (2011). Subgrouping patients with low back pain: a treatment-based approach to classification. Sports Health.

[26] Walker BF, Williamson OD (2009). Mechanical or inflammatory low back pain: What are the potential signs and symptoms?. Man Ther.

[27] Petersen T, Laslett M, Thorsen H, Manniche C, Ekdahl C, Jacobsen S (2003). Diagnostic classification of non-specific low back pain. A new system integrating patho-anatomic and clinical categories. Physiother Theory Pract.

[28] Hensley CP, Courtney CA (2014). Management of a patient with chronic low back pain and multiple health conditions using a pain mechanisms-based classification approach. J Orthop Sports Phys Ther.

[29] Yusuf S, Wittes J, Probstfield J, Tyroler HA (1991). Analysis and interpretation of treatment effects in subgroups of patients in randomized clinical trials. JAMA.

[30] Deutsche Schmerzgesellschaft e.V. (2018). Deutscher Schmerzfragebogen.

[31] Gerbershagen HU, Zimmermann M, Klingler D, Morawetz R, Thoden U (1996). Das Mainzer Stadienkonzept des Schmerzes: Eine Standortbestimmung. Antidepressiva als Analgetika.

[32] Frettlöh J, Maier C, Gockel H, Zenz M, Hüppe M (2009). Patientenkollektiv deutscher schmerztherapeutischer Einrichtungen: Kerndaten von mehr als 10.000 Patienten. Schmerz.

[33] Frettlöh J, Maier C, Gockel H, Hüppe M (2003). Validität des Mainzer Stadienmodells der Schmerzchronifizierung bei unterschiedlichen Schmerzdiagnosen. Schmerz.

[34] Hampel P, Moergel MF (2009). Schmerzchronifizierung bei Rückenschmerzpatienten in der stationären Rehabilitation: Zur Validität des Mainzer Stadienmodells der Schmerzchronifizierung. Schmerz.

[35] Schmitt N, Gerbershagen HU (1990). The Mainz pain staging system (MPSS) for chronic pain. Pain.

[36] Hüppe M, Maier C, Gockel H, Zenz M, Frettlöh J (2011). Behandlungserfolg auch bei höherer Schmerzchronifizierung? Eine Auswertung des Mainzer Stadienmodells auf Basis der QUAST-Analysestichprobe. Schmerz.

[37] Von Korff M, Ormel J, Keefe FJ, Dworkin SF (1992). Grading the severity of chronic pain. Pain.

[38] Klasen BW, Hallner D, Schaub C, Willburger R, Hasenbring M (2004). Validation and reliability of the German version of the Chronic Pain Grade questionnaire in primary care back pain patients. Psychosoc Med.

[39] Bjelland I, Dahl AA, Haug TT, Neckelmann D (2002). The validity of the Hospital Anxiety and Depression Scale: An updated literature review. J Psychosom Res.

[40] Herrmann C (1997). International experience with the Hospital Anxiety and Depression Scale. A review of validation data and clinical results. J Psychosom Res.

[41] White D, Leach C, Sims R, Atkinson M, Cottrell D (1999). Validation of the Hospital Anxiety and Depression Scale for use with adolescents. Br J Psychiatry.

[42] Snaith RP (2003). The Hospital Anxiety And Depression Scale. Health Qual Life Outcomes.

[43] Herrmann-Lingen C, Buss U, Snaith RP (2011). HADS-D – Hospital Anxiety and Depression Scale Deutsche Version.

[44] Lovibond PF, Lovibond SH (1995). The structure of negative emotional states: comparison of the Depression Anxiety Stress Scales (DASS) with the Beck Depression and Anxiety Inventories. Behav Res Ther.

[45] Nilges P, Essau C (2015). Die Depressions-Angst-Stress-Skalen: Der DASS – ein Screeningverfahren nicht nur für Schmerzpatienten. Schmerz.

[46] Basler HD (1999). Marburger Fragebogen zum habituellen Wohlbefinden – Untersuchung an Patienten mit chronischem Schmerz. Schmerz.

[47] Basler HD, Herda C, Scharfenstein A, Schumacher J, Klaiberg A, Brähler E (2003). Marburger Fragebogen zum habituellen Wohlbefinden. Diagnostische Verfahren zu Lebensqualität und Wohlbefinden.

[48] Ware J, Kosinski M, Keller SD (1996). A 12-Item Short-Form Health Survey: construction of scales and preliminary tests of reliability and validity. Med Care.

[49] Ware JE, Spilker B (1996). The SF-36 Health Survey. Quality of life and pharmacoeconomics in clinical trials.

[50] Korb J, Pfingsten M (2003). Der Deutsche Schmerzfragebogen – Implementierte Psychometrie. Schmerz.

[51] Holm K (2018). ALMO Statistiksystem 15.

[52] Bacher J (2010). Clusteranalyse: Anwendungsorientierte Einführung in Klassifikationsverfahren.

[53] IBM Software Group (2018). IBM SPSS Statistics Base 22.

[54] Gockel HH, Maier C (2000). QUAST – Auswertungsorientiertes EDV-System zur Dokumentation und Qualitätssicherung in der Schmerztherapie. Schmerz.

[55] Gesetz über die Entgelte für voll- und teilstationäre Krankenhausleistungen (Krankenhausentgeltgesetz – KHEntgG): § 21 Übermittlung und Nutzung von Daten.

[56] International Association for the Study of Pain (2018). Pain treatment Services.

[57] Kaufman L, Rousseeuw PJ (2005). Finding Groups in Data: An Introduction to Cluster Analysis.

[58] Fritz JM, Cleland JA, Childs JD (2007). Subgrouping patients with low back pain: evolution of a classification approach to physical therapy. J Orthop Sports Phys Ther.

[59] Solovieva S, Leino-Arjas P, Saarela J, Luoma K, Raininko R, Riihimäki H (2004). Possible association of interleukin 1 gene locus polymorphisms with low back pain. Pain.

[60] Tegeder I, Costigan M, Griffin RS, Abele A, Belfer I, Schmidt H, Ehnert C, Nejim J, Marian C, Scholz J, Wu T, Allchorne A, Diatchenko L, Binshtok AM, Goldman D, Adolph J, Sama S, Atlas SJ, Carlezon WA, Parsegian A, Lötsch J, Fillingim RB, Maixner W, Geisslinger G, Max MB, Woolf CJ (2006). GTP cyclohydrolase and tetrahydrobiopterin regulate pain sensitivity and persistence. Nat Med.

[61] Karppinen J, Solovieva S, Luoma K, Raininko R, Leino-Arjas P, Riihimäki H (2009). Modic changes and interleukin 1 gene locus polymorphisms in occupational cohort of middle-aged men. Eur Spine J.

[62] Costigan M, Belfer I, Griffin RS, Dai F, Barrett LB, Coppola G, Wu T, Kiselycznyk C, Poddar M, Lu Y, Diatchenko L, Smith S, Cobos EJ, Zaykin D, Allchorne A, Gershon E, Livneh J, Shen PH, Nikolajsen L, Karppinen J, Männikkö M, Kelempisioti A, Goldman D, Maixner W, Geschwind DH, Max MB, Seltzer Z, Woolf CJ (2010). Multiple chronic pain states are associated with a common amino acid-changing allele in KCNS1. Brain.

[63] Turk DC, Rudy TE (1988). Toward an empirically derived taxonomy of chronic pain patients: integration of psychological assessment data. J Consult Clin Psychol.

[64] Turk DC (2005). The potential of treatment matching for subgroups of patients with chronic pain: lumping versus splitting. Clin J Pain.

[65] Dahlström L, Widmark G, Carlsson SG (1997). Cognitive-behavioral profiles among different categories of orofacial pain patients: diagnostic and treatment implications. Eur J Oral Sci.

[66] Turk DC, Okifuji A (1998). Treatment of chronic pain patients: Clinical outcomes, cost-effectiveness, and cost-benefits of multidisciplinary pain centers. Crit Rev Phys Rehabil Med.

[67] Johansson E, Lindberg P (2000). Low back pain patients in primary care: Subgroups based on the multidimensional pain inventory. Int J Behav Med.

[68] Turk DC, Okifuji A (2002). Psychological factors in chronic pain: evolution and revolution. J Consult Clin Psychol.

[69] Söderlund A, Denison E (2006). Classification of patients with whiplash associated disorders (WAD): reliable and valid subgroups based on the Multidimensional Pain Inventory (MPI-S). Eur J Pain.

[70] Thieme K, Rose U, Pinkpank T, Spies C, Turk DC, Flor H (2006). Psychophysiological responses in patients with fibromyalgia syndrome. J Psychosom Res.

[71] Widerström-Noga EG, Felix ER, Cruz-Almeida Y, Turk DC (2007). Psychosocial subgroups in persons with spinal cord injuries and chronic pain. Arch Phys Med Rehabil.

[72] Heidari J, Mierswa T, Hasenbring M, Kleinert J, Levenig C, Belz J, Kellmann M (2018). Recovery-stress patterns and low back pain: Differences in pain intensity and disability. Musculoskeletal Care.

[73] Huijnen IP, Verbunt JA, Peters ML, Smeets RJ, Kindermans HP, Roelofs J, Goossens M, Seelen HA (2011). Differences in activity-related behaviour among patients with chronic low back pain. Eur J Pain.

[74] McCracken LM, Samuel VM (2007). The role of avoidance, pacing, and other activity patterns in chronic pain. Pain.

[75] Hasenbring MI, Hallner D, Klasen B, Streitlein-Böhme I, Willburger R, Rusche H (2012). Pain-related avoidance versus endurance in primary care patients with subacute back pain: psychological characteristics and outcome at a 6-month follow-up. Pain.

[76] Foster NE (2011). Barriers and progress in the treatment of low back pain. BMC Med.

[77] Kamper SJ, Apeldoorn AT, Chiarotto A, Smeets RJ, Ostelo RW, Guzman J, van Tulder MW (2014). Multidisciplinary biopsychosocial rehabilitation for chronic low back pain. Cochrane Database Syst Rev.

[78] Wettstein M, Eich W, Bieber C, Tesarz J (2019). Profiles of Subjective Well-being in Patients with Chronic Back Pain – Contrasting Subjective and Objective Correlates. Pain Med.

[79] Viniol A, Jegan N, Hirsch O, Leonhardt C, Brugger M, Strauch K, Barth J, Baum E, Becker A (2013). Chronic low back pain patient groups in primary care – a cross sectional cluster analysis. BMC Musculoskelet Disord.

[80] Boersma K, Linton SJ (2005). Screening to identify patients at risk: profiles of psychological risk factors for early intervention. Clin J Pain.

[81] Hill JC, Whitehurst DG, Lewis M, Bryan S, Dunn KM, Foster NE, Konstantinou K, Main CJ, Mason E, Somerville S, Sowden G, Vohora K, Hay EM (2011). Comparison of stratified primary care management for low back pain with current best practice (STarT Back): a randomised controlled trial. Lancet.

[82] Fehrmann E, Tuechler K, Kienbacher T, Mair P, Spreitzer J, Fischer L, Kollmitzer J, Ebenbichler G (2017). Comparisons in Muscle Function and Training Rehabilitation Outcomes Between Avoidance-Endurance Model Subgroups. Clin J Pain.

[83] Hasenbring M, Klasen B, Rusu AC, Hasenbring MI, Rusu AC, Turk DC (2012). Risk factor-based cognitive-behavioural therapy for acute and subacute back pain. From Acute to Chronic Back Pain: Risk Factors, Mechanisms, and Clinical Implications.

[84] Morrison DG (1967). Measurement problems in cluster analysis. Management Science.

